# Clinical application of enhanced recovery after surgery in bipolar transurethral vaporization of prostate for benign prostatic hyperplasia

**DOI:** 10.3389/fsurg.2025.1702376

**Published:** 2025-10-23

**Authors:** Xinliang Cai, Yuan Yuan, Lin Jiang, Yifei Zhu, Ruidong Ji, Mingchi Wang, Ruxi Tang, Zhenquan Lu, Bingfeng Luo

**Affiliations:** 1 Division of Urology, Department of Surgery, The University of Hong Kong -Shenzhen Hospital, Shenzhen, China; 2 The First Clinical Medical College of Jinan University, Guangzhou, China

**Keywords:** enhanced recovery after surgery, bipolar transurethral vaporization of the prostate, length of stay, hospitalization costs, postoperative complications

## Abstract

**Objective:**

To explore the clinical application of enhanced recovery after surgery (ERAS) in bipolar transurethral vaporization of the prostate (B-TUVP) and its impact on postoperative recovery.

**Methods:**

A retrospective analysis was conducted on the clinical data of 212 patients with benign prostatic hyperplasia (BPH) admitted to the Department of Urology of our hospital from January 2019 to December 2024. Among them, 121 patients were managed under an ERAS protocol (ERAS group), and 91 patients received traditional perioperative care (control group). The clinical characteristics included age, mean body mass index (BMI), preoperative comorbidities, prostate volume (PV), preoperative International Prostate Symptom Score (IPSS), preoperative Quality of Life (QoL) score, preoperative urinary flow rate, and urodynamic parameters. Preoperative management, intraoperative management, and postoperative outcomes were compared between the two groups.

**Results:**

The clinical baseline data showed no significant differences between the two groups. Compared with the control group, the ERAS group had a significantly shorter duration of continuous bladder irrigation, time to first ambulation, and postoperative length of stay (*P* < 0.001). The total hospitalization costs were also significantly lower in the ERAS group compared to the control group (*P* < 0.01). There was no significant difference in the incidence of postoperative complications between the two groups.

**Conclusion:**

The application of the ERAS protocol in B-TUVP can promote postoperative recovery, reduce the postoperative length of stay, and decrease total hospitalization costs. However, more prospective randomized controlled trials are needed to further validate the feasibility and effectiveness of this protocol.

## 1 Introduction

The concept of enhanced recovery after surgery (ERAS) was first introduced by the Danish surgeon Kehlet in 1997 and has since been rapidly adopted and promoted in Western countries (1). Currently, well-established ERAS clinical pathways have been developed and implemented across various surgical disciplines, including gastrointestinal surgery (2), gynecology (3), and orthopedics (4). Within the field of urology, ERAS has been widely applied in procedures such as adrenalectomy (5), nephrectomy (6), radical cystectomy (7), and radical prostatectomy (8). Numerous studies have confirmed its advantages in reducing hospital length of stay, lowering hospitalization costs, decreasing postoperative complications, and improving patient quality of life.

However, research on the application of ERAS in surgery for benign prostatic hyperplasia (BPH) remains relatively limited. A small number of retrospective studies have indicated that ERAS protocols can shorten the duration of postoperative catheterization and reduce pain scores in BPH patients (9, 10). These studies provided preliminary evidence for the safety and efficacy of ERAS in transurethral surgery for BPH, although no statistically significant differences were reported in total hospitalization costs or overall postoperative complication rates between groups (11). However, clinical research on the application of the ERAS concept in bipolar transurethral vaporization of the prostate (B-TUVP) is limited. This study aims to compare the ERAS protocol (ERAS group) with traditional perioperative care (control group) to evaluate its feasibility, safety, and impact on postoperative recovery indicators in patients undergoing B-TUVP.

## 2 Materials and methods

A retrospective analysis was conducted on the clinical data of 212 patients with BPH who were admitted to the Department of Urology of our hospital between January 2019 and December 2024. Among them, 91 patients treated from January 2019 to December 2021 were assigned to the traditional care group (control group), while 121 patients treated from January 2022 to December 2024 were managed under an ERAS protocol (ERAS group). The clinical characteristics of the two groups, including age, mean body mass index (BMI), preoperative comorbidities, prostate volume (PV), preoperative International Prostate Symptom Score (IPSS), preoperative Quality of Life (QoL) score, and preoperative urinary flow rate, showed no statistically significant differences (*P* > 0.05).

Inclusion criteria were as follows: ① age between 55 and 85 years; ② PV between 30 and 200 ml; ③ patients with inadequate response to pharmacological therapy who met the surgical indications for BPH. Exclusion criteria were as follows: ① PV < 30 ml; ② PV > 200 ml; ③ history of urethral stricture; ④ concurrent bladder stones or bladder diverticula requiring surgical intervention; ⑤ severe cardiopulmonary diseases, coagulation disorders, or organ dysfunction (e.g., severe hepatic or renal insufficiency); ⑥ acute infectious diseases.

All patients underwent thorough preoperative preparation to achieve the following criteria: ① patients with hypertension required pharmacological control of blood pressure to 120/80 mmHg and heart rate to 80–90 beats per minute; ② negative urine cultures prior to surgery; ③ for diabetic patients, fasting blood glucose was maintained below 7 mmol/L and random blood glucose below 11 mmol/L; ④ stable electrolyte and acid-base balance; ⑤ completion of IPSS and QoL assessments; ⑥ venous thromboembolism risk assessment for all patients, with anticoagulation therapy administered based on the assessed risk.

## 3 Perioperative management protocols

### 3.1 Preoperative management

ERAS Group: Patients received education on the ERAS concept; fasting was required for 6 h and clear fluid intake was prohibited for 4 h preoperatively; no bowel preparation was administered; anti-embolism stockings were applied one day before surgery. Control Group: Patients received routine preoperative education; fasting was required for 8 h and clear fluid intake was prohibited for 6 h preoperatively; conventional bowel preparation was administered; anti-embolism stockings were not used.

### 3.2 Intraoperative management

ERAS Group: A single dose of prophylactic antibiotics was administered 30 min before surgery; mechanical thromboprophylaxis was applied during the procedure; forced-air warming blankets were used for thermal insulation. Control Group: A single dose of prophylactic antibiotics was administered 30 min before surgery; no mechanical thromboprophylaxis was applied; no active warming measures were used.

### 3.3 Anesthesia and surgical technique

**Figure 1:**
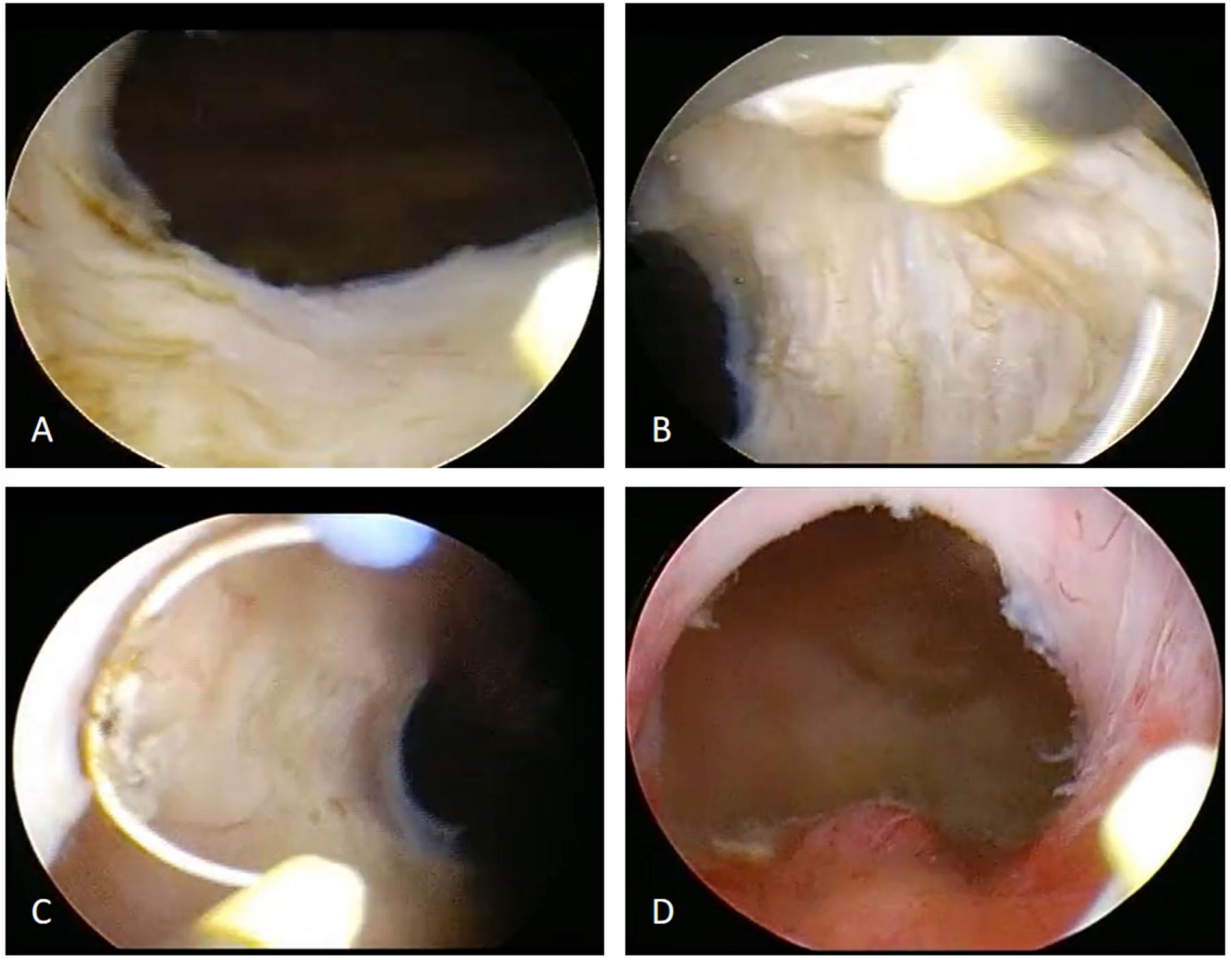
Surgical flowchart of bipolar transurethral vaporization of the prostate (B-TUVP). **(A)** The median lobe of the prostate was resected to the surgical capsule. **(B)** The left lobe was then resected to the surgical capsule. **(C)** The right lobe was subsequently resected to the surgical capsule. **(D)** Finally, the tissue at the 12 o'clock position and the apical prostate were carefully trimmed. After achieving meticulous hemostasis, inspection of the prostatic fossa at the verumontanum confirmed a satisfactory outlet and an intact external urethral sphincter.

Both the ERAS and control groups received either spinal anesthesia or general anesthesia with endotracheal intubation. All procedures were performed by the same senior surgeon using a standardized surgical approach and resection technique: namely the resection of three-lobe technique was used (Figure 1). Briefly, channels were first created at the 5 and 7 o'clock positions down to the surgical capsule of the prostate. The median lobe was resected firstly, followed by the left lateral lobe and then the right lateral lobe. Finally, the tissue at the 12 o'clock position and the apical prostate were carefully trimmed. After achieving meticulous hemostasis, inspection of the prostatic fossa at the verumontanum confirmed a satisfactory outlet and an intact external urethral sphincter. The resected prostate tissues were irrigated out and sent for pathological examination.

### 3.4 Postoperative management

ERAS Group: Patients were administered 500∼1,000 mL of intravenous fluids postoperatively and commenced oral water intake 6 h after surgery. Control Group: Patients received 1,000∼2,000 mL of intravenous fluids postoperatively and began oral water intake 8 h after surgery.

### 3.5 Outcome measures

The following parameters were rigorously documented: duration of continuous bladder irrigation, time to first ambulation, time to catheter removal, length of hospital stay, total hospitalization costs, and incidence of postoperative complications. Laboratory values, including hemoglobin, potassium and sodium levels, were measured on the first postoperative day. The IPSS and uroflowmetry results were evaluated at three months postoperatively.

### 3.6 Statistical analysis

All statistical analyses were performed using SPSS software (version 25.0). Continuous data were assessed for normality using the Shapiro–Wilk test and for homogeneity of variances using Levene's test. Normally distributed data are presented as mean ± standard deviation and were compared using the independent samples *t*-test. Non-normally distributed data are presented as median (interquartile range) and were compared using the Mann–Whitney *U*-test. Categorical data were analyzed using the Chi-square test or Fisher's exact test, as appropriate. A two-sided *P*-value < 0.05 was considered statistically significant. Given the retrospective nature of this study, an *a priori* power calculation was not performed. A *post hoc* power analysis using G*Power software indicated that with the present sample sizes (*n* = 121 vs. *n* = 91), an alpha of 0.05, and a power of 0.8, the smallest detectable effect size (Cohen's d) for continuous variables was 0.39. For outcomes analyzed with *t*-tests, the observed effect sizes (Cohen's d) for length of stay (d ≈ 0.59) and hospitalization costs (d ≈ 0.43) exceeded this threshold. For outcomes analyzed with non-parametric tests, effect sizes (r) were calculated accordingly. This supports the detectability of these key differences.

## 4 Results

A total of 212 patients (121 in the ERAS group and 91 in the control group) were included in the final analysis. The baseline clinical characteristics were comparable between the two groups. The mean age was 69.74 ± 7.44 years in the ERAS group and 70.75 ± 7.47 years in the control group (*p* = 0.335). The mean BMI was 21.36 ± 3.00 kg/m^2^ in the ERAS group, with no significant difference compared to the control group (*p* = 0.084). There was no statistically significant difference in baseline comorbidities between the two groups. Further analyses of PV, preoperative IPSS, preoperative QoL score, and urinary flow rate also showed no statistically significant differences (*p* > 0.05). Detailed clinical data are summarized in Table 1. Intraoperative and postoperative outcomes, including complications, were compared between the two groups (Table 2). The ERAS group showed a significantly shorter duration of continuous bladder irrigation, earlier time to first ambulation, and reduced postoperative length of stay compared to the control group (*p* < 0.001). Total hospitalization costs were also significantly lower in the ERAS group (*p* = 0.004). However, no significant difference was observed in the time to catheter removal (*p* = 0.731). No significant intergroup differences were found in hemoglobin, potassium and sodium levels on postoperative day 1 (Table 3). The incidence of postoperative complications, included bleeding (defined as a hemoglobin dro*p* < 70 g/L), procedure-related fever, urinary tract infection, deep vein thrombosis, and re-admission, did not differ significantly between groups (Table 3). All patients successfully underwent the minimally invasive procedure without major complications (Clavien-Dindo Grade IV) or mortality.

**Table 1:** Clinical data of the patients in both groups.

Parameters	Control group （*n* = 91)	ERAS group （*n* = 121)	*P-value*
Age/year	69.74 ± 7.44	70.75 ± 7.47	0.335
BMI (kg/m^2^)	23.94 ± 3.56	23.16 ± 3.00	0.084
Basic disease, %(n)			
Diabetes mellitus	11.0%（10/91）	16.5%（20/121）	0.246
Hypertension	51.6%（47/91）	38.0%（46/121）	0.034
Coronary heart disease	14.3%（13/91）	14.0%（17/121）	0.955
Others	47.3%（43/91）	43.8%（53/121）	0.720
PV（mL）	66.24 ± 32.13	69.75 ± 34.26	0.451
Preoperative IPSS	20.80 ± 4.98	21.60 ± 4.00	0.212
Peroperative QOL score	4.70 ± 0.80	4.66 ± 0.85	0.755
Preoperative urinary flow rate（Qmax, ml/s）	6.23 ± 3.32	5.36 ± 3.06	0.051
Urodynamic parameters.（Pdet, cmH2O）	73.17 ± 28.08	63.03 ± 33.36	0.018

Data are presented as mean ± standard deviation or % (n); ERAS, enhanced recovery after surgery; BMI, body mass index; PV, prostate volume; IPSS, international prostate symptom score; QoL, quality of life; *Pdet,* detrusor pressure; Qmax: maximum urinary flow rate.

**Table 2:** Preoperative, intraoperative and postoperative management.

Parameters	Control group （*n* = 91)	ERAS group （*n* = 121)	*P-value*
Preoperative Management			
Education on the ERAS concept	No	Yes	NA
Bowel preparation	Yes	No	NA
Fasting before surgery (h)	8	6	NA
Drinking before surgery (h)	6	4	NA
Intraoperative Management			
Surgical approach	B-TUVP	B-TUVP	NA
Prophylactic antibiotics	A single dose of prophylactic antibiotics was administered 30 min before surgery	A single dose of prophylactic antibiotics was administered 30 min before surgery	NA
Intraoperative mechanical thromboprophylaxis	No	Yes	NA
Intraoperative forced-air warming blankets	No	Yes	NA
Postoperative Management			
Duration of continuous bladder irrigation (h)	27.91 ± 14.89	21.18 ± 5.16	<0.001
Time to first ambulation (h)	28.16 ± 12.75	21.04 ± 5.00	<0.001
Time to urinary catheter removal (h)	113.28 ± 44.50 （4.72 day）	115.44 ± 36.86（4.81 day）	0.713
Postoperative length of stay (day)	6.31 ± 3.32	4.88 ± 1.47	<0.001
Total hospitalization costs (CNY)	19,159.22 ± 10,786.63	15,878.88 ± 3,603.53	0.004

Data are presented as mean ± standard deviation or % (n); ERAS: enhanced recovery after surgery; B-TUVP: bipolar transurethral vaporization of the prostate; CNY: Chinese Yuan.

**Table 3:** Analysis of postoperative laboratory parameters, IPSS, urinary flow rate and complications in two groups.

Parameters	Control group （*n* = 91)	ERAS group （*n* = 121)	*P-value*
Hemoglobin postoperative day 1(g/L)	128.26 ± 17.54	128.47 ± 14.63	0.923
Potassium postoperative day 1 (mmol/L)	3.98 ± 0.36	3.99 ± 0.40	0.837
Sodium postoperative day 1 (mmol/L)	141.33 ± 2.34	140.83 ± 2.29	0.128
Postoperative complications			
Bleeding	4.4%（4/91）	0.8%（1/121）	0.167
Procedure-related fever	6.6%（6/91）	3.3%（4/121）	0.324
Urinary tract infection	8.8%（8/91）	9.9%（12/121）	0.781
Deep vein thrombosis	3.3%（3/91）	0.8%（1/121）	0.316
Re-admission	2.2%（2/91）	4.1%（5/121）	0.458
Postoperative IPSS	7.90 ± 2.37	7.40 ± 2.17	0.629
Postoperative urinary flow rate (Qmax, mL/s）	15.55 ± 5.33	16.68 ± 4.94	0.112

Data are presented as mean ± standard deviation or % (n); ERAS: enhanced recovery after surgery; IPSS: international prostate symptom score; Qmax: maximum urinary flow rate.

## 5 Discussion

ERAS protocols are designed to attenuate the physiological and psychological stress of surgery, minimize postoperative complications, and promote faster recovery, thereby facilitating earlier discharge (1). The successful implementation of ERAS requires a standardized, multidisciplinary approach involving surgeons, anesthesiologists, nurses, nutritionists, and physical therapists. In addition to its application in gastrointestinal (11), pancreatic (12), cardiac (13), gynecological (3), and orthopedic surgery (4), ERAS protocols have been increasingly adopted in urological procedures. These include surgeries for renal tumors (6), radical cystectomy (7), radical prostatectomy (8), and adrenalectomy (5, 14). Current evidence indicates that ERAS protocols not only accelerate postoperative recovery but also shorten hospital stay, reduce hospitalization costs, and improve patient satisfaction, contributing to more efficient utilization of healthcare resources.

However, clinical studies on the application of the ERAS concept in transurethral surgery for BPH remain limited. Zhou et al. (9) conducted a study involving 486 patients (248 in the ERAS group and 238 in the conventional group), reporting on the clinical implementation of an ERAS protocol in transurethral prostate surgery. With a follow-up period of one year, the results demonstrated that the ERAS group had a significantly shorter catheterization time (1.0 day vs. 2.7 days, *P* < 0.01) and markedly lower postoperative pain scores (*P* < 0.01). However, there were no significant differences in postoperative complications or hospitalization costs between the two groups. Similarly, no statistically significant differences were observed in IPSS and QoL scores within one year after surgery. These findings support the feasibility and safety of implementing ERAS protocols in transurethral surgery for BPH. An et al. (10) compared ERAS-based rapid bladder irrigation with conventional irrigation in patients undergoing transurethral resection of the prostate or holmium laser enucleation of the prostate. ERAS-guided irrigation enabled faster clearance of tissue debris and blood, resulting in clearer effluent, earlier catheter removal, and shorter hospitalization, without increasing postoperative bleeding or other complications. The authors concluded that ERAS-based irrigation is safe, effective, and cost-efficient.

Therefore, the above findings indicate that the ERAS protocol not only facilitates earlier removal of the urinary catheter but also reduces hospitalization costs, thereby alleviating the financial burden on patients and providing overall clinical benefits. In this study, we further evaluated the feasibility and safety of applying ERAS principles to B-TUVP. The protocol was integrated across all perioperative phases, including the preoperative, intraoperative, and postoperative phases. Preoperatively, patients received structured education and counseling to reduce anxiety and improve adherence. To minimize prolonged fasting and dehydration, a modified protocol (6 h for solids, 4 h for clear fluids) was adopted, which helped reduce intraoperative hypoglycemia, insulin resistance, and hemodynamic instability. No bowel preparation was performed, reducing unnecessary fluid loss and preventing rectal mucosal injury. Intraoperatively, warming devices were used to maintain normothermia, an intervention particularly important for elderly patients with cardiovascular comorbidities. Prevention of hypothermia decreases stress-induced catecholamine release, reduces the risk of myocardial ischemia and arrhythmia, and lowers intraoperative blood loss and surgical site infection. Mechanical thromboprophylaxis was employed as part of a multimodal antithrombotic strategy, complementing pharmacologic prophylaxis, early ambulation, ankle pump exercises, temperature control, and goal-directed fluid therapy. Postoperatively, early oral fluid intake (within 6 h) and ankle pump exercises were encouraged to facilitate gastrointestinal recovery and reduce venous thromboembolism risk. Consequently, patients in the ERAS group demonstrated significantly shorter durations of continuous bladder irrigation (21.18 ± 5.16 vs. 27.91 ± 14.89 h, *P* < 0.001) and earlier ambulation (21.04 ± 5.00 vs. 28.16 ± 12.75 h, *P* < 0.001) compared to the control group.

Bae et al. (15) demonstrated in a cohort of 427 patients undergoing GreenLight HPS laser photoselective vaporization of the prostate that catheter removal on the first postoperative day was both safe and feasible, without increasing complications. Similarly, Tam et al. (16) highlighted that, within a structured multidisciplinary ERAS protocol for abdominal surgery, early catheter removal is a viable strategy that does not elevate the risk of postoperative urinary retention. In our center, the conventional practice following B-TUVP has been catheter removal between postoperative days 3 and 6. Prolonged catheterization may increase the risk of pain, bladder spasms, or urinary tract infection, whereas early removal raises concerns about bleeding or acute urinary retention. In our study, no statistically significant difference was observed in catheterization time between the ERAS and conventional groups (4.81vs.4.72 days, *P* = 0.713). Furthermore, rates of postoperative bleeding, urinary tract infection, and other complications were comparable (all *P* > 0.05), and no Clavien–Dindo grade IV complications occurred in either group.

The application of ERAS principles in colorectal surgery has been shown to accelerate recovery, shorten hospital stays, reduce complications, and provide cost-effectiveness. Consistent with these observations, Yan et al. (17) reported that ERAS protocols in laparoscopic adrenalectomy reduced disposable material use, medication, and diagnostic testing costs, yielding a mean saving of ¥4,416 per patient and underscoring the economic advantage of ERAS adoption. Our findings similarly indicate that the ERAS group undergoing B-TUVP experienced a significantly shorter hospital stay (4.88 ± 1.47 vs. 6.31 ± 3.32 days, *P* < 0.001) and lower total hospitalization costs (¥15,878.88 ± 3,603.53 vs. ¥19,159.22 ± 10,786.63, *P* < 0.001), highlighting both clinical and economic benefits.

Infection prevention was uniformly applied as a cornerstone of perioperative care. All patients received a single preoperative antibiotic dose, followed by a 24-h postoperative course. No significant differences were observed in infection-related complication rates between the groups. Nevertheless, it is crucial to interpret this finding in the context of the study's limited statistical power for dichotomous outcomes. Assuming a baseline event rate of 10%, the minimal detectable absolute difference was approximately 14.4%. Thus, the lack of statistical significance may reflect a Type II error rather than true equivalence, necessitating caution in interpretation and warranting future larger-scale studies to robustly compare safety outcomes. Regarding other secondary endpoints, electrolyte levels (sodium and potassium) and hemoglobin on postoperative day 1 showed no significant intergroup differences. Furthermore, functional outcomes, including IPSS and urinary flow rate, were comparable at the 3-month follow-up.

This study, while focused on B-TUVP, ultimately underscores the transcendent value of the ERAS philosophy in urological surgery. The rapid evolution of surgical technologies for BPH, notably the rise of laser vaporization and enucleation, should not overshadow the critical importance of perioperative management. The significant improvements we observed in recovery metrics and cost-effectiveness were achieved not by changing the surgical technology, but by systematically implementing a multidisciplinary, evidence-based care pathway. This suggests that the core principles of ERAS—preoperative counseling, stress reduction, and early mobilization—are universally applicable and can be synergistically combined with any surgical technique, whether established or novel, to maximize patient benefit and healthcare value.

## 6 Conclusion

The implementation of an ERAS protocol in patients undergoing B-TUVP was associated with significant clinical benefits, including reduced bladder irrigation time, earlier ambulation, shorter postoperative length of stay, and lower hospitalization costs, without increasing perioperative complications. These outcomes support the safety, feasibility, and cost-effectiveness of the ERAS approach in B-TUVP, indicating its suitability for broader clinical adoption. Nevertheless, several limitations of this study should be acknowledged. First, as a retrospective analysis conducted at a single institution, the findings may be influenced by unmeasured confounders and selection bias. Second, with the ongoing advancement of surgical techniques for BPH, further investigation is needed to determine whether ERAS confers consistent benefits across different operative modalities. Third, continued research is warranted to refine and personalize perioperative ERAS strategies for patients undergoing B-TUVP. Future prospective, multi-institutional randomized controlled trials are necessary to validate the generalizability of these findings and to further establish the role of ERAS protocols in B-TUVP and other transurethral procedures.

## Data Availability

The original contributions presented in the study are included in the article/Supplementary Material, further inquiries can be directed to the corresponding authors.
